# Maternal Serum Vitamin B12 during Pregnancy and Offspring Autism Spectrum Disorder

**DOI:** 10.3390/nu15082009

**Published:** 2023-04-21

**Authors:** Andre Sourander, Sanju Silwal, Heljä-Marja Surcel, Susanna Hinkka-Yli-Salomäki, Subina Upadhyaya, Ian W. McKeague, Keely Cheslack-Postava, Alan S. Brown

**Affiliations:** 1 Research Centre for Child Psychiatry, INVEST Flagship, University of Turku, 20014 Turku, Finland; 2 Department of Child Psychiatry, Turku University Hospital, 20521 Turku, Finland; 3 Faculty of Medicine, University of Oulu, 90014 Oulu, Finland; 4 Biobank Borealis of Northern Finland, Oulu University Hospital, 90014 Oulu, Finland; 5 Department of Biostatistics, Columbia University Mailman School of Public Health, New York, NY 10032, USA; 6 Department of Psychiatry, New York State Psychiatric Institute, Columbia University Irving Medical Center, New York, NY 10032, USA; 7 Department of Epidemiology, Columbia University Mailman School of Public Health, New York, NY 10032, USA

**Keywords:** maternal vitamin B12, autism spectrum disorder, ASD, maternal, prenatal

## Abstract

This study examined the association between maternal serum vitamin B12 levels during early pregnancy and offspring autism spectrum disorders (ASD) and subtypes. Based on a Finnish national birth cohort, case offspring *(n* = 1558) born in 1987–2007 and diagnosed with ASD by 2015 were matched with one control on date of birth, sex and place of birth. Maternal vitamin B12 levels were measured during first and early second trimesters of pregnancy. High maternal vitamin B12 levels (≥81th percentile) was associated with increased risk for offspring childhood autism, adjusted odds ratio, 1.59, 95% confidence interval 1.06–2.41 (*p* = 0.026). No significant associations were observed between maternal vitamin B12 levels and offspring Asperger’s or pervasive developmental disorder/NOS.

## 1. Introduction

Autism Spectrum Disorder (ASD) is a heterogeneous group of neurodevelopmental conditions characterized by impairment in social communication and interaction, accompanied by restricted and repetitive patterns in behaviors, interests and activities [1]. There is a wide recognition that the etiology of ASD is multifactorial, involving the interplay of genetic and environmental factors [2]. Strong evidence has suggested that ASD is influenced by prenatal factors [3] including maternal nutrition [4]. Specific nutrients have been identified as potentially impacting the risk for ASD, for example, folate, vitamin D, iron and polyunsaturated fatty acids, but the findings are often mixed [5,6]. 

Vitamin B12 is essential for normal growth, development and physiological functions [7]. It plays an important role in neural myelination, synaptogenesis and neurotransmitter synthesis [8]. Since the fetus is completely dependent on maternal nutrients, inadequate vitamin B12 levels during pregnancy may impair these processes and cause neural damage or brain atrophy [9,10]. Experimental rodent studies have shown a link between maternal folate and vitamin B12 deficiency with structural brain abnormalities [11], alteration in gene expression in the cerebellum [12,13] and cognitive impairment in offspring [14,15]. In humans, folate and vitamin B12 deficiency have been associated with poor neurodevelopment [16] and negative effects on cognitive functions including memory, language and motor skills [17,18,19], preterm birth, low birth weight [20] and insulin resistance [21].

As shown in Table S1, prior studies on maternal vitamin B12 and offspring ASD were mainly focused on multivitamin or folate supplementation with inconsistent findings. Only one study specifically examined the association between maternal plasma vitamin B12 and offspring ASD [22]. The authors showed that high vitamin B12 levels (≥536.8 pmol/L) increased the risk for offspring ASD. Of note, the study included only 86 ASD cases and maternal blood samples were collected 24–72 h post-delivery. Four studies examined the association between maternal plasma folate and offspring ASD or autistic traits, but the findings were inconsistent [22,23,24,25]. Two of these studies, one from Sweden (N = 100) and the other from the USA (N = 86) showed high levels of maternal plasma folate associated with increased risk of ASD in offspring [22,24]. However, two other studies from the Netherlands (N = 3893) and the USA (N = 209) did not observe any associations between maternal folate concentration and child autistic traits [23,25]. The use of folate and/or multivitamins early or during pregnancy showed no associations, increased or decreased risk for ASD or ASD symptoms in offspring [26,27,28,29,30,31,32,33,34,35,36,37]. 

There is a lack of knowledge from the existing literature about the role of maternal vitamin B12 as a possible risk factor for offspring ASD. First, no previous study has examined maternal serum vitamin B12 levels during pregnancy and offspring risk for ASD diagnosis. Previous studies on maternal vitamin B12 and offspring ASD were based on multivitamin supplementation, and the composition of the multivitamins used during pregnancy was unclear [26,27,28,29,30,31,32,33,34,35,36,37]. Second, out of three studies examining associations between maternal folate concentrations during early pregnancy and offspring ASD [23,24,25], two studies were based on ASD symptoms [23,25] and one study was on ASD diagnosis [24]. 

The aim of this population-based study was to examine the association between maternal serum vitamin B12 levels in early pregnancy and the risk of ASD diagnosis in offspring. In addition, we investigated the association between maternal serum vitamin B12 levels in early pregnancy and specific ASD subtypes in the offspring. Based on the findings of maternal folate and vitamin B12 deficiency associated with impaired cognitive behavior in rodent offspring [14], we hypothesized that lower maternal vitamin B12 levels would be associated with an increased risk of offspring ASD. This would have important public health implications for preventative strategies. Since vitamin B12 is found in animal-sourced foods, vitamin B12 deficiency has been widely prevalent in those with poor intake of animal origin foods owing to the religious or cultural practice of vegetarianism [38]. This could be readily preventable by supplementation. 

## 2. Materials and Methods

The Finnish Prenatal Study of Autism Spectrum Disorders (FIPS-A) is a nested case-control study including all singleton live births between 1987–2005 in Finland. Each child was followed for the diagnosis of ASD to the end of 2015. The data for this study were derived from three national registers: the Care Register for Health Care (CRHC), the Finnish Medical Birth Register (FMBR) and the Finnish Population Register Centre (FCPR). These registers are linked using personal identity codes that are issued at birth or on immigration to all Finnish residents since 1964. 

### 2.1. Nationwide Registers 

The CRHC includes all public and private inpatient diagnoses since 1967 and all outpatient diagnoses from specialized services since 1998. The diagnostic classification in Finland is based on the International Classification of Diseases (ICD): ICD-8 from 1969 to 1986, ICD-9 from 1987 to 1995 and ICD-10 since 1996. A previous diagnostic validation study of ASD has shown 96% specificity for childhood autism [39]. The FMBR contains nationwide comprehensive data on all live births during the neonatal period up to 7 days of age since 1987. The FCPR is a computerized national archive maintained by the Finnish population center and local register officers. It contains basic demographic information (for example, name, personal identity codes, address, citizenship and native language, family relations, date of birth and death (if applicable)) of current Finnish citizens and permanent residents in Finland dating back to 1969. The FCPR was used to identify the controls and to obtain information on the subjects’ parents and places of birth. 

The study was conducted in accordance with the Declaration of Helsinki, and FIPS-A received ethical approval from the Ethics Committee of the Hospital District of Southwest Finland(Dnro number 24/007), the data protection authorities at the National Institute for Health and Welfare and the Institutional Review Board of the New York State Psychiatric Institute (5740). The study design and the request for biobank serum samples were approved by the responsible Biobank’s Scientific Committee (Biobank Borealis of Northern Finland, University of Oulu, Oulu, Finland) (BB_2017_1017). 

### 2.2. Finnish Maternity Cohort 

The Finnish Maternity Cohort of the Northern Finland Biobank Borealis (FMC) is a nationwide serum bank that consists of approximately 2 million serum samples collected during the first and early second trimesters of pregnancy (5th to 95th percentile: months 2–4 of pregnancy) from over 950,000 women. After informed consent, the remaining serum samples (one sample of 1–3 mL for each pregnancy) were stored at −25 °C in a protected biorepository at the Biobank Borealis in Oulu, Finland and were available for scientific research. All samples in the FMC were linked with offspring and other Finnish nationwide registers by a unique personal identification code that has been issued to each resident of Finland since 1971. 

### 2.3. Information on Cases and Controls

The ASD cases were born in Finland between January 1987 and December 2007 and were registered in the CRHC with ICD-10 (F84x) and ICD-9 (299x) diagnoses by 2015. Controls were singleton offspring born in Finland and without a diagnosis of ASD or Intellectual disability (ID). Each case was matched with one control on date of birth (+/−30 days), sex and place of birth. The controls were alive at the time of the diagnosis of the matched cases. During 1987–2005, there were 4705 cases with diagnoses of childhood autism, Asperger’s and PDD/NOS. In total, 1558 cases and 1558 matched controls were included in the study. The subjects with ID were identified from CRHC with ICD codes: ICD-10 (F70, F71, F72, F73, F78, and F79) and ICD-9 (317, 318.0, 318.1, 318.2, and 319).

### 2.4. Maternal Vitamin B12 Measurement

To examine maternal vitamin B12 levels in the prenatal sera, we measured active B12 (HoloTC, Holotranscobalamin) using a chemiluminescence microparticle immunoassay on the Architect i2000SR automatic immunoassay analyzer (Abbott Diagnostics). The coefficient of variation (mean ± SD) derived from repeated quality control samples included in each set of daily assays was 4.7% in the control samples with high B12 levels (range 43.7–49.6 pmol/L) and 6.4% in those with low B12 levels (range 13.6–17.3 pmol/L).

### 2.5. Covariates

Potential confounders and mediators suggested to be associated with both maternal vitamin B12 and ASD were selected [40,41,42,43,44]. The FMBR was used to obtain information on the number of previous births, maternal socioeconomic status (SES), maternal age, maternal smoking during pregnancy, gestational age, Apgar score at 1 min and weight for gestational age. As smokers are known to have lower vitamin B12 levels [45,46], maternal smoking during pregnancy was included in the covariate testing. Information on maternal and paternal psychiatric diagnoses and maternal substance abuse diagnoses was obtained from the CRHC. Maternal immigrant status was obtained from the FCPR, while gestational week and season of blood draw were obtained from the FMC. The variables included in the analysis were categorized as follows: maternal smoking (yes, no), previous births (0, ≥1), history of maternal psychopathology (yes, no), history of paternal psychopathology (yes, no), maternal SES (upper white collar, lower white collar, blue collar, others, missing), history of maternal substance abuse (yes, no), gestational age (<37 years, ≥37 years), weight for gestational age (<−2 SD, −2SD to +2SD, > + 2SD), maternal immigration status (yes, no), season of blood collection (spring, summer, autumn, winter) and Apgar score (0–6, 7–8, 9–10). Detailed descriptions of covariates are given in Table 1 and Table 2.

### 2.6. Statistical Analysis 

Maternal vitamin B12 levels were initially examined as a continuous variable. Since vitamin B12 levels had a skewed distribution, they were log-transformed before analysis. We also examined maternal vitamin B12 levels categorized into quintiles. The cut-off of the quintiles for case and control groups was based on the distribution of maternal vitamin B12 levels in the control group, with the third quintile as the reference group. Continuous potential confounders were tested with χ^2^, Student’s t and Fisher’s exact tests and/or Pearson chi-square tests for categorical variables for the association with log-transformed maternal vitamin B12 levels among controls. Potential confounders were then tested for association with ASD using conditional logistic regression models for the matched sets. The covariates were included in the adjusted models based on their association with both the exposure and the outcome at *p* < 0.1. 

The point and interval estimate of odds ratios (OR) were obtained by fitting conditional logistic regression models for matched pairs. Unadjusted ORs and adjusted odds ratios (aORs) and 95% confidence intervals (CI) were calculated separately for ASD and 3 subgroups: childhood autism, Asperger’s and Pervasive Developmental Disorder (PDD)/PDD-not otherwise specified (NOS). A sensitivity analysis was performed adjusting for gestational age as it is a risk factor for ASD (Allen et al., 2020). Further sensitivity analysis was performed additionally adjusting for maternal psychopathology, substance abuse, and offspring gestational age to address prior evidence that maternal alcohol use affects vitamin B12 and folate levels (Laufer et al., 2004). Additional analyses were performed stratifying ASD with and without ID. Conditional logistic regression was used to test for interactions between continuous maternal vitamin B12 and ASD by subgroups of ASD, sex, timing of gestational week of blood draw and gestational age. Statistical significance was based on 2-sided *p* < 0.05. All statistical analyses were performed with SAS 9.4 software (SAS 9.4, SAS Institute, Cary, N.C., USA).

## 3. Results

The study included 1558 ASD case-controlled pairs. The mean age of ASD diagnosis for cases was 6.57 years (SD: 3.21, Range: 0–18 years). The median vitamin B12 level among cases was 114.40 pmol/L, and it was 116.80 pmol/L among controls. The mean gestational week of maternal blood draw was 10.79 weeks (SD 3.5) for cases and 10.10 weeks (SD 2.8) for controls. The gender distribution was 19.5% female and 80.5% male in cases and controls.

Among potential covariates in the study, gestational week of blood draw, maternal SES and maternal immigration status were associated with maternal vitamin B12 levels among controls with *p* < 0.1 (Table 1). Maternal age, gestational week of blood draw, previous births, maternal psychopathology, paternal psychopathology, maternal SES, maternal substance abuse, gestational age, weight for gestational age and Apgar score were associated with offspring ASD with *p* < 0.1 (Table 2). Maternal SES and gestational week of blood draw were associated with both maternal vitamin B12 levels and offspring ASD and were adjusted in multivariable models. 

Table 3 shows the log-transformed maternal serum vitamin B12 levels and offspring ASD and their subcategories. Maternal serum vitamin B12 was not associated with offspring ASD in either unadjusted (OR 0.90, 95% CI 0.77–1.06, *p* = 0.209) or adjusted analyses (aOR 0.94, 95% CI 0.79–1.10, *p* = 0.441). In a subsequent analysis of ASD subtypes, no significant associations were observed between maternal serum vitamin B12 levels and ASD subtypes in unadjusted or adjusted analyses (Table 3).

**Figure 1:**
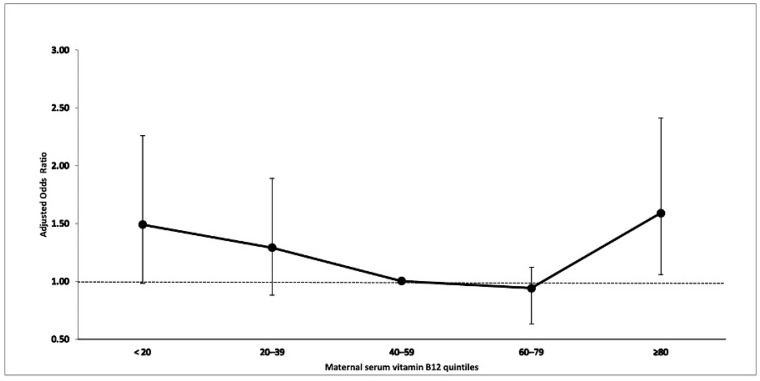
Association between maternal serum vitamin B12 and offspring childhood autism.

Table 4 shows the distribution of maternal vitamin B12 levels in quintiles by case-control status. A significant association was observed between maternal vitamin B12 levels in the highest quintile (≥81th percentile) and offspring childhood autism (aOR 1.59, 95% CI 1.06–2.41, *p* = 0.026) and the association was close to significance in the lowest quintile (aOR 1.49, 95% CI 0.98–2.26, *p* = 0.064), relative to the third quintile (Figure 1). No significant association was observed between maternal vitamin B12 levels and ASD, the Asperger’s subgroup or the PDD/PDD NOS subgroup. 

In additional sensitivity analyses, we adjusted for offspring gestational age for the association between log-transformed maternal serum vitamin B12 levels or measured in quintiles, and offspring ASD and their subcategories. Furthermore, we additionally adjusted for maternal psychopathology (history of psychopathology and substance abuse) and gestational age (see Supplemental Tables S2 and S3). The findings were similar with significant association between childhood autism and maternal vitamin B12 in the highest quintile in model I (aOR 1.59, 95% CI 1.06–2.42, *p* = 0.027) and in model II (aOR 1.75, 95% CI 1.14–2.67, *p* = 0.010). No significant association was observed between maternal vitamin B12 levels and ASD, the Asperger’s subgroup or the PDD/PDD NOS subgroup.

In further additional analysis, we stratified the sample by ASD with and without ID and examined the association with maternal vitamin B12 levels. No significant association was observed between log-transformed maternal vitamin B12 levels and offspring ASD with ID (aOR 1.06, 95% CI 0.69–1.64, *p* = 0.796) or without ID (aOR 0.92, 95% CI 0.77–1.10, *p* = 0.369) (see Supplemental Table S4). 

Testing for the sex and maternal vitamin B12 interaction did not reveal evidence for a modification effect by sex on the relationship between continuous maternal vitamin B12 and ASD (*p* = 0.492). We found no interaction between continuous maternal vitamin B12 and gestational week of blood draw (*p* = 0.437), and also no interaction was observed between continuous maternal vitamin B12 and gestational age (*p* = 0.334). 

## 4. Discussion

This is the first population-based study examining maternal vitamin B12 levels in prenatal sera in relation to ASD. We found high levels of maternal vitamin B12 (≥81th percentile) during early pregnancy associated with offspring childhood autism. These findings are consistent with the study by Raghavan et al. [22] of 86 ASD cases that showed vitamin B12 in the highest decile (≥90th percentile) was associated with an increased risk of ASD in offspring. However, maternal blood samples were collected 24–72 h post-delivery. Furthermore, in the current study, low levels of maternal vitamin B12 (<20th percentile) during early pregnancy evidenced a statistical trend toward association with offspring childhood autism. While interesting, this result should be interpreted with caution. One possible explanation for this finding could be that vitamin B12 follows Bertrand’s rule, as do other micronutrients [47]. Bertrand’s rule describes the dose–response curve for many micronutrients as non-monotonic, with benefits increasing with intake at low levels, then plateauing with optimal concentrations and finally, toxicity occurring at higher levels. However, it is important to note that these findings were observed only in childhood autism and we do not know of a specific mechanism that would produce this pattern of association. More studies are needed to confirm these associations. 

We did not find any associations between maternal vitamin B12 levels in early pregnancy and offspring Asperger’s syndrome or PDD/PDD-NOS. Since the etiology of ASD is multifactorial, different etiologic factors may have different levels of association with specific subgroups within the ASD spectrum. Of note, in a nested case-controlled study, maternal serum vitamin B12 levels in early pregnancy were not associated with offspring attention-deficit hyperactivity disorder [48]. In a study by Brown et al. [49], high homocysteine levels (which are inversely related to vitamin B12, as well as folate) were associated with an increased risk for schizophrenia among offspring. 

Previous studies on humans and animal have shown that folic acid and vitamin B12 deficiency during pregnancy can cause persistent changes in the offspring’s genome, and resulting in brain abnormalities that are associated with ASD [12,13,50]. The key developmental genes involved in neural pathways showed altered expression and impairment in many of the pathways that are linked to ASD [51,52]. Vitamin B12 also plays an important role in deoxyribonucleic acid methylation, cellular growth and differentiation [53,54]. However, very little is known about the role of maternal vitamin B12 on human brain development. While our findings are observational and do not address the mechanisms behind the association, we believe that a potential role for high levels of maternal vitamin B12 may have some role in the etiology of childhood autism, the most severe phenotype of ASD, and this deserves future research. 

The strengths of this study include the use of archived biospecimens for the measurement of maternal serum vitamin B12 during pregnancy, a large sample size and the capacity to adjust for a large number of confounders. There are several limitations that should be considered. First, ASD diagnoses included only children referred to specialized services and are likely to represent the most severe ASD cases. The findings of the study will not represent mild and moderate forms of ASD. Second, we did not measure neonatal vitamin B12 levels. However, the maternal and neonatal serum levels of vitamin B12 have been shown to significantly correlate with one another [55,56,57]. Third, maternal vitamin B12 levels in this study were collected in the first and early second trimesters of pregnancy. The findings thus cannot be generalized to B12 levels occurring in later pregnancy. Fourth, residual confounding by unmeasured factors (such as maternal body mass index, prenatal vitamin supplementation or maternal medications during pregnancy) could be possible. 

## 5. Conclusions

The present study revealed high maternal vitamin B12 levels (≥81th percentile) during early pregnancy were associated with offspring childhood autism. The study raises questions on the impact of very extremely elevated levels of maternal vitamin B12 levels on early brain development. If these findings are confirmed in future studies, it would indicate that maternal vitamin B12 has specificity as an etiological factor for a severe form of ASD and high maternal vitamin B12 levels have toxic effects on offspring. Further studies may have potential for clinical relevance to identify optimal levels of maternal vitamin B12 supplementation during pregnancy. 

## Figures and Tables

**Table 1:** Relationship between covariates and maternal vitamin B12 levels (≥/< median) among controls.

Covariates	Maternal Vitamin B12		Maternal Vitamin B12			*p*-Value
	≥Median		<Median			
	Mean	SD	Mean	SD		
					**t**	
**Maternal age (years)**	29.35	5.23	29.69	5.08	1.30	0.195
**Gestational week of blood draw**	9.94	2.75	10.26	2.87	2.31	0.021
	**n**	**%**	**n**	**%**	**χ^2^**	
**Maternal smoking**					1.78	0.409
No	659	84.38	636	81.85		
Yes	107	13.70	123	15.83		
**Previous births ^1^**						
0	315	40.38	332	43.01	1.09	0.295
≥1	465	59.62	440	56.99		
**History of maternal** **Psychopathology ^a^**					0.07	0.795
No	674	86.30	667	85.84		
Yes	107	13.70	110	14.16		
**History of paternal psychopathology ^b 2^**					0.04	0.834
No	676	86.89	668	86.53		
Yes	102	13.11	104	13.47		
**Maternal SES**					8.32	0.081
Upper white collar	107	13.70	117	15.06		
Lower white collar	329	42.13	293	37.71		
Blue collar	161	20.61	148	19.05		
Others	116	14.85	122	15.70		
Missing	68	8.71	97	12.48		
**History of maternal substance abuse ^c^**					1.16	0.282
No	762	97.57	751	96.65		
Yes	19	2.43	26	3.35		
**Gestational age (weeks) ^3^**					2.53	0.112
<37	20	2.57	31	4.01		
≥37	759	97.43	743	95.99		
**Weight for gestational age ^4^**					3.91	0.141
<−2 SD	13	1.67	15	1.94		
−2 SD to +2 SD	733	94.09	739	95.60		
>+2 SD	33	4.24	19	2.46		
**Maternal immigration status**					7.41	0.007
No	760	97.31	735	94.59		
Yes	21	2.69	42	5.41		
**Season of blood collection**					3.23	0.358
Spring	191	24.46	196	25.23		
Summer	212	27.14	186	23.94		
Autumn	198	25.35	192	24.71		
Winter	180	23.05	203	26.13		
**Apgar Score ^5^**						
0–6	27	3.47	30	3.88	0.72	0.699
7–8	164	21.11	151	19.53		
9–10	586	75.42	592	76.58		

SES, socioeconomic status; SD, standard deviation; t: *t*-test value; χ^2^: Pearson’s chi squared test value. ^a^ ICD-8 (291–308), ICD-9 (291–316) or ICD-10 (F10–99, excluding maternal substance abuse diagnosis; ^b^ ICD-8 (291–308), ICD-9 (291–316) or ICD-10 (F10–99); ^c^ ICD-8 (291, 303, 304), ICD-9 (291, 292, 303, 304, 305) or ICD-10 (F10–19) ^1^ Data missing for 6 controls; ^2^ Data missing for 8 controls; ^3^ Data missing for 6 controls; ^4^ Data missing for 6 controls; ^5^ Data missing for 8 controls.

**Table 2:** Relationship between covariates and ASD in case and control subjects.

Covariates	Cases		Controls			*p*-Value
	Mean	SD	Mean	SD		
					**t**	
**Maternal age (years)**	29.91	5.45	29.53	5.16	−2.00	0.046
**Gestational week of blood draw**	10.79	3.53	10.09	2.81	−6.05	<0.001
	**n**	**%**	**n**	**%**	**χ^2^**	
**Maternal smoking**					2.25	0.325
No	1273	81.71	1295	83.12		
Yes	240	15.40	230	14.76		
**Previous births ^1^**					12.41	<0.001
0	746	47.97	647	41.69		
≥ 1	809	52.03	905	58.31		
**History of maternal psychopathology ^a^**					91.44	<0.001
No	1124	72.14	1341	86.07		
Yes	434	27.86	217	13.93		
**History of paternal psychopathology ^b 2^**					36.19	<0.001
No	1201	78.50	1344	86.71		
Yes	329	21.50	206	13.29		
**Maternal SES**					9.83	0.043
Upper white collar	197	12.64	224	14.38		
Lower white collar	599	38.45	622	39.92		
Blue collar	288	18.49	309	19.83		
Others	297	19.06	238	15.28		
Missing	177	11.36	165	10.59		
**History of maternal substance abuse ^c^**					13.39	<0.001
No	1472	94.48	1513	97.11		
Yes	86	5.52	45	2.89		
**Gestational age (weeks) ^3^**					15.11	<0.001
<37	97	6.26	51	3.28		
≥37	1453	93.71	1502	96.72		
**Weight for gestational age ^4^**					7.68	0.022
<−2 SD	52	3.36	28	1.80		
−2 SD to +2 SD	1441	93.03	1472	94.85		
>+2 SD	56	3.62	52	3.35		
**Maternal immigration status**					0.14	0.712
No	1499	96.21	1495	95.96		
Yes	59	3.79	63	4.04		
**Season of blood collection**					82.91	0.406
Spring	350	22.46	387	24.84		
Summer	411	26.38	398	25.55		
Autumn	416	26.70	390	25.03		
Winter	381	24.45	383	24.58		
**Apgar Score ^5^**						
0–6	72	4.63	57	3.68	4.94	0.085
7–8	353	22.72	315	20.32		
9–10	1129	72.65	1178	76.00		

SES, socioeconomic status; SD, standard deviation; t: *t*-test value; χ^2^: Pearson’s chi squared test value. Cases and Controls are matched date of birth (+/−30 days), sex and place of birth ^a^ ICD-8 (291–308), ICD-9 (291–316) or ICD-10 (F10–99, excluding maternal substance abuse diagnosis; ^b^ ICD-8 (291–308), ICD-9 (291–316) or ICD-10 (F10–99); ^c^ ICD-8 (291, 303, 304), ICD-9 (291, 292, 303, 304, 305) or ICD-10 (F10–19) ^1^ Data missing for 3 cases, 6 controls; ^2^ Data missing for 28 cases, 8 controls; ^3^ Data missing for 8 cases, 5 controls; ^4^ Data missing for 9 cases, 6 controls; ^5^ Data missing for 4 cases, 8 controls.

**Table 3:** Odds ratios and 95% CIs for the association between log-transformed maternal serum vitamin B12 (continuous) and offspring ASD and subtypes.

Log-Transformed Maternal Vitamin B12 Levels (pmol/L)	Case (N = 1558)	Control (N = 1558)	Odds Ratio Unadjusted (95% CI)	*p*-Value	Odds Ratio Adjusted ^a^ (95% CI)	*p*-Value
	Median	Median				
**ASD**	4.74	4.74	0.90 (0.77–1.06)	0.209	0.94 (0.79–1.10)	0.441
**Childhood autism**	**(n = 491)**	**(n = 491)**				
	4.78	4.75	0.94 (0.71–1.25)	0.660	0.96 (0.72–1.28)	0.774
**Asperger’s**	**(n = 536)**	**(n = 536)**				
	4.74	4.74	0.96 (0.72–1.27)	0.759	1.05 (0.78–1.42)	0.734
**PDD/PDD-NOS**	**(n = 531)**	**(n = 531)**				
	4.73	4.75	0.83 (0.64–1.08)	0.169	0.87 (0.66–1.15)	0.316

ASD, autism spectrum disorder; CI, confidence interval; PDD, Pervasive Developmental Disorder; PDD-NOS, PDD-not otherwise specified. ^a^ Adjusted for gestational week of blood draw and maternal socioeconomic status.

**Table 4:** Odds ratios and 95% CIs for the association between maternal serum vitamin B12 levels (in quintiles) and offspring ASD and subtypes.

Maternal Serum Vitamin B12 in Quintiles (pmol/L)	Cases n (%)	Controls n (%)	Odds Ratio Unadjusted (95% CI)	*p*-Value	Odds Ratio Adjusted ^a^ (95% CI)	*p*-Value
1. **ASD**	**(N = 1558)**	**(N = 1558)**				
0–20 (0–80.8)	331 (21.25)	312 (20.03)	1.13 (0.91–1.41)	0.278	1.06 (0.85–1.34)	0.582
21–40 (80.9–105.4)	328 (21.05)	313 (20.09)	1.12 (0.89–1.39)	0.331	1.09 (0.87–1.36)	0.455
41–60 (105.5–128.9)	290 (18.61)	310 (19.90)	Reference		Reference	
61–80 (128.8–164.9)	310 (19.90)	312 (20.03)	1.06 (0.85–1.33)	0.618	1.09 (0.87–1.36)	0.853
≥81 (≥165)	299 (19.19)	311 (19.96)	1.02 (0.82–1.28)	0.849	1.02 (0.81–1.29)	0.869
2. **Childhood autism**	**(N = 491)**	**(N = 491)**				
0–20 (0–80.8)	104 (21.18)	86 (17.52)	1.52 (1.01–2.31)	0.047	1.49 (0.98–2.26)	0.064
21–40 (80.9–105.4)	99 (20.16)	94 (19.14)	1.27 (0.87–1.86)	0.212	1.29 (0.88–1.89)	0.199
41–60 (105.5–128.9)	90 (18.33)	111 (22.61)	Reference		Reference	
61–80 (128.8–164.9)	89 (18.33)	111 (22.61)	0.99 (0.67–1.49)	0.997	0.94 (0.63–1.42)	0.778
≥81 (≥165)	109 (22.20)	89 (18.13)	1.52 (1.02–2.28)	0.042	1.59 (1.06–2.41)	0.026
3. **Asperger’s**	**(N = 536)**	**(N = 536)**				
0–20 (0–80.8)	110 (20.52)	120 (22.39)	0.97 (0.68–1.39)	0.872	0.87 (0.60–1.27)	0.477
21–40 (80.9–105.4)	114 (21.27)	109 (20.34)	1.13 (0.77–1.66)	0.528	1.03 (0.69–1.53)	0.883
41–60 (105.5–128.9)	98 (18.28)	103 (19.22)	Reference		Reference	
61–80 (128.8–164.9)	116 (21.64)	97 (18.10)	1.29 (0.87–1.90)	0.205	1.25 (0.83–1.87)	0.283
≥81 (≥165)	98 (18.28)	107 (19.96)	0.98 (0.67–1.43)	0.901	0.99 (0.67–1.47)	0.965
4. **PDD/PDD-NOS**	**(N = 531)**	**(N = 531)**				
0–20 (0–80.8)	117 (22.03)	106 (19.96)	1.04 (0.70–1.54)	0.854	0.99 (0.65–1.51)	0.965
21–40 (80.9–105.4)	115 (21.66)	110 (20.72)	0.99 (0.68–1.45)	0.954	0.99 (0.66–1.49)	0.980
41–60 (105.5–128.9)	102 (19.21)	96 (18.08)	Reference		Reference	
61–80 (128.8–164.9)	105 (19.77)	104 (19.59)	0.95 (0.64–1.39)	0.789	0.99 (0.66–1.50)	0.982
≥81 (≥165)	92 (17.33)	115 (21.66)	0.76 (0.52–1.12)	0.168	0.74 (0.49–1.12)	0.159

ASD, autism spectrum disorder; CI, confidence interval; PDD, PDD, Pervasive Developmental Disorder; PDD-NOS, PDD-not otherwise specified. ^a^ Adjusted for gestational week of blood draw and maternal socioeconomic status.

## Data Availability

Deidentified individual participant data will not be made available due to ethical restrictions. Summary level data can be obtained from the corresponding author upon reasonable request.

## Supplementary Materials

The following supporting information can be downloaded at https://www.mdpi.com/article/10.3390/nu15082009/s1, Table S1: Review of previous studies on maternal vitamin B12 and multivitamin and autism spectrum disorders, by primary method of exposure assessment; Table S2. Odds ratios and 95% CIs for the association between log-transformed maternal serum vitamin B12 (continuous) and offspring ASD and subtypes adjusting for additional covariates; Table S3: Odds ratios and 95% CIs for the association between maternal serum vitamin B12 levels (in quintiles) and offspring ASD and subtypes adjusting for additional covariates; Table S4. Odds ratios and 95% CI of the association between log-transformed maternal serum vitamin B12 (continuous, quintiles) and offspring Autism with and without Intellectual Disability (ID).
