# Supplementary material for: Maternal Serum Vitamin B12 during Pregnancy and Offspring Autism Spectrum Disorder
Source: Nutrients. 2023 Apr 21;15(8):2009. doi: 10.3390/nu15082009 (PMC10146734; doi:10.3390/nu15082009)
Supplement: Supplementary file 1 [file nutrients-15-02009-s001.zip › nutrients-2228365-supplementary.pdf]

Maternal serum vitamin B12 during pregnancy and offspring Autism Spectrum Disorder

Andre Sourander [1,2,*+], Sanju Silwal [1+], Heljä-Marja Surcel [3,4], Susanna Hinkka-Yli-Salomäki [1], Subina Upadhyaya [1], Ian W. McKeague [5], Keely Cheslack-Postava [6], Alan S. Brown [6,7]

[1] Research Centre for Child Psychiatry, INVEST Flagship, University of Turku, 20014 Turku, Finland
[2] Department of Child Psychiatry, Turku University Hospital, 20521 Turku, Finland
[3] Faculty of Medicine, University of Oulu, 90014 Oulu, Finland
[4] Biobank Borealis of Northern Finland, Oulu University Hospital, 90014 Oulu, Finland
[5] Department of Biostatistics, Columbia University Mailman School of Public Health, New York, NY 10032, USA
[6] Department of Psychiatry, New York State Psychiatric Institute, Columbia University Irving Medical Center, New York, NY 10032, USA
[7] Department of Epidemiology, Columbia University Mailman School of Public Health, New York, NY 10032, USA
* Correspondence: andsou@utu.fi; Tel.: +358-50-365-3447 or +358-2-333-7668 or +358-2-333-7394
† These authors contributed equally and share equal first authorship to this work.

Supplement

Table S1: Review of previous studies on maternal vitamin B12 and multivitamin and autism spectrum disorders, by primary method of exposure assessment

**Author/ Country/**	**Diag nosis**	**Study design**	**Diagnosti c criteria**	**Measures of exposure**	**Dosage**	**Sample size/Age range**	**Covariates**	**Results, Effect size estimate and 95% CI**
				**Biomarker studies**				
Raghavan et al., 2018<br>USA	ASD	Prospective cohort, Clinical Boston Medical Center Birth	ICD-9	Maternal blood samples collected 24-72 hours post-delivery<br>Self-reported maternal multivitamin supplement use	NA	ASD, n=86<br>TD, n=1171	Child: gestational age, sex and year of birth<br>Mother: age, education, parity, BMI, smoking status, diabetes status, race and MTHFR genotype	Plasma B_{12} (>600 pmol/L), HR 3.0 (1.6-5.7); ref (≥200-≤600 pmol/L)<br>Plasma B12 (≥90th percentile), HR 2.5 (1.4-4.5), ref ((≥10th to <90thpercentile)<br>Plasma folate at birth (≥60.3 nmol/L), HR 2.5 (1.3-4.6), ref (>14.7 to <60.3)<br>Multivitamin use: First trimester<br>≤ 2/week HR 3.4 (1.6, 7.2), >5/week HR 2.3 (1.2, 3.9)<br>Second trimester<br>≤ 2/week HR 3.8 (1.8, 8.0), >5/week HR 2.1 1.2, 3.6<br>Third trimester<br>≤ 2/week HR 3.5 (1.7, 7.4), >5/week HR 2.1 1.2, 3.6<br>ref: multivitamins 2–5 times/week
Egorova et al., 2020<br>Sweden	ASD	Case-control	ICD-10	Maternal serum folate concentrations at 14 weeks of pregnancy	NA	ASD, n=100 (74-infantile autism, 26-Asperger syndrome)<br>TD, n=100	Child: sex<br>Maternal: age at sampling, serum cotinine and 5-methyl tetrahydropholate levels<br>Other: year of sampling	Folate, nmol/L<br>OR 1.70 (1.22-2.37)

Steenweg-de Graaff et al., 2014 Netherlands	Autistic traits	Population-based cohort, Generation R	SRS, CBCL-Parent reported	Maternal plasma folate concentration: median 13.2 weeks of gestation (10.5-17.2 weeks)<br>FA use from preconception to 10 weeks of pregnancy	NA	Autistic traits, n=3893	Child: age, sex, gestational age at venipuncture	FA before conception<br>OR -0.04 (-0.07 to -0.02),<br>FA first 10 weeks of pregnancy,<br>OR -0.04 (-0.07 to -0.02)<br>FA after first 10 weeks of pregnancy,<br>OR -0.06 (-0.09 to -0.03)<br>ref no use<br>Folate concentrations, nmol/L<br>B -0.004 (-0.013 to 0.004)
Braun et al., 2014 USA	Autistic behaviors	Population-based cohort, HOME	SRS-Mother report	Whole blood folate: 11-21 weeks gestation<br>Prenatal vitamin use: 14-39 weeks gestation	NA	Autistic traits, n=209	Maternal: age, race, education, prenatal vitamin use, marital status, employment insurance status, depressive symptoms, serum cotinine concentrations<br>food security, fresh fruit/vegetable intake<br>Household: income	Prenatal vitamin weekly/daily<br>OR 0.26 (0.08-0.89), ref no use<br><br>Folate concentration, nmol/L<br>OR 1.42 (0.81-2.49)
**Record based**								
Moser et al., 2019 Israel	ASD	Nested case-control	DSM	Dispensing record for FA with or without multivitamin supplements one year prior to child's birth	Cumulative FA dose[a]	ASD, n=2009<br>TD, n=19,886	Child: sex, birth year, birth order<br>Mother: age, region of residence, poverty index, subfertility, number of family physician and obstetric obgyn visits, diabetes mellitus, hypertension, cardiovascular disease, cancer,<br>epilepsy, antifolate medications[b]	FA supplement, ref ≤0.2 mg/day<br>0.2-<0.4mg/day<br>OR 1.27 (0.98-1.65)<br>0.4-<1mg/day<br>OR 1.10 (0.98-1.24)<br>1-<3mg/day<br>OR 1.14 (0.98-1.34)<br>≥3mg/day<br>OR 1.01 (0.60-1.70)

Levine et al 2018 Israel	ASD	Case-control cohort	ICD-9	Pharmacy records, multivitamins and /or FA supplements before and during pregnancy	NA	ASD, n=572 Controls, n=45300	Child: sex, birth year Maternal: parity, SES (high vs low), psychiatric diagnosis at childbirth, age Paternal psychiatric diagnosis and age	FA and/or multivitamin supplements, ref no exposure Before pregnancy (540-271 days before childbirth), RR, 0.39 (0.30-0.50) During pregnancy (270 days before childbirth up to the date of childbirth), RR, 0.27 (0.22-0.33), FA before pregnancy, RR, 0.56 (0.42-0.74) FA during pregnancy, RR, 0.32 (0.26-0.41) Multivitamin before pregnancy, RR 0.36 (0.24-0.52) Multivitamin during pregnancy, RR 0.35 (0.28-0.44)
**Self-report**								
Brieger et al., 2021	ASD	Prospective cohort of high risk families, EARLI	DSM	Multivitamin, prenatal and folic acid intake during pregnancy	FA (<400mcg, 400-1000mcg, >1000mcg) in one month	ASD, n=38 Non-ASD, n=153	Child: sex Maternal: education	Prenatal vitamin use, ref no use OR 0.70 (0.32-1.53) Folic acid, ref 400-1000 mcg <400 mcg OR 0.75 (0.32-1.69) >1000 mcg OR 1.64 (0.44-5.47)
Tan et al., 2020 China	ASD	Case-control	DSM	FA supplementation 12 weeks before and 12 weeks after LMP Micronutrient supplementation from LMP to birth	FA: 400µg daily Micronutrient: NA	ASD, n=416 TD, n=201	Child; age, sex, gestational age, birth weight Maternal; residence (rural/urban), labor mode Paternal; age Household: income	No FA supplementation, OR 1.91 (1.24-2.93), No micronutrient supplements OR 1.721 (1.19-2.47) ref any supplement use
DeVilbiss et al 2017 Sweden	ASD	Prospective cohort study (Stockholm Youth cohort)	ICD 10 DSM-IV-TR	Self-reported multivitamin use during pregnancy	NA	ASD without ID sibling cohort, n=173209	Child: sex, birth year, years reside in Stockholm County, socio-economic indicators (education,	Multivitamin use ASD, OR 0.89 (0.82-0.97) ASD with ID, OR 0.69 (0.57-0.84) ASD without ID, OR 0.94 (0.85-1.03)

						ASD sibling cohort, n=174428<br>ASD with ID, n=169760	family income, maternal birth country)<br>Maternal:(age, BMI, parity, smoking status, medications (antidepressants or antiepileptics), neuropsychiatric conditions[c]	Folic acid supplement<br>ASD, OR 1.27 (1.01-1.60)<br>ASD without ID, OR 1.29 (0.99-1.67)<br>ASD with ID, OR 1.20 (0.71-2.01)
Surén et al, 2013<br>Norway	ASD	Prospective cohort MoBa	ADI-R<br>ADOS<br>DSM-IV<br>ICD-10	Self-reported multivitamin, FA supplement 4 weeks before LMP and 8 weeks after LMP	NA	ASD, n=114<br>85,176	Child: birth year<br>Maternal: planned pregnancy, smoking, BMI, parity, weight gain at 18 and 30 weeks<br>Parental: education, age	FA, OR 0.61 (0.41–0.90), ref no supplement use
Nilsen et al., 2013<br>Norway	ASD	Population based cohort, MoBA	ICD-10	Self-reported FA intake<br>Before and/or during pregnancy	NA	ASD, n=234	Child; birth year<br>Maternal; age, marital status, parity, hospital size<br>Paternal; age	FA<br>OR 0.86 (0.78, 0.95), ref no supplement use
**Interview**								
Schmidt et al. 2019<br>USA	ASD	Prospective cohort of high risk families MARBLES	MSEL<br>ADOS at 3 years of age	Vitamin and supplement use[d] 6 months preconception and each month during pregnancy	NA	Siblings of children with ASD-total, n=241<br>ASD, n=55<br>TD, n=126<br>Non-TD, n=60	Child: birthplace, sex, year of birth<br>Maternal: education, age, pre-pregnancy BMI, planned pregnancy, race/ethnicity, home ownership, insurance delivery type	Prenatal vitamin<br>RR 0.50 (0.30-0.81), ref, no supplement use<br>Folic acid supplement<br>≥600 μg/day RR 0.51 (0.31-0.82), ref < 600 μg/day
Li et al 2018<br>China	ASD	Retrospective case-control	SRS<br>DSM-IV	FA supplement use and food preference in preconception and during pregnancy assessed 3–6 years after delivery	NA	ASD, n=374<br>TD, n=354	Child: age, premature birth, sex<br>Maternal: pre-pregnancy BMI and delivery, premature	FA before pregnancy<br>OR 0.95 (0.61-1.50)<br>FA during pregnancy<br>OR 0.64 (0.41-1.00)<br>ref, no supplement use

							delivery, intake of other supplements<br>Parental age and education	
Strom et al. 2018<br>Denmark	ASD	Prospective cohort<br>DNBC	ICD-10	FA use and multivitamin:4-8 weeks of gestation and mid gestation, 25 weeks gestation	FA use<br>0, <400, ≥400 µg/day	ASD, n=12 34<br>Childhood autism, n=312	Child: sex<br>Maternal: age, parity, smoking, education, SES, planned pregnancy, pre-pregnancy BMI<br>Paternal: age	Any FA, ref no supplement use<br>At GW 4- 8, HR 1.06, (0 .94- 1.19),<br>Mid pregnancy,<br><400 µg/day, HR 1.01 (0.76-1.34)<br>400 µg/day, HR 0.98 (CI 0.75-1.29)<br>Folate from food<br>Q2: HR 0.82 (0.67, 1.01),<br>Q3: HR 0.96 (0.78, 1.17),<br>Q4: HR 0.85 (0.69, 1.04)<br>Q5: HR 0.94 (0.77, 1.16) (ref: Q1 quintile)
Virk et al 2016<br>Denmark	ASD	Prospective cohort<br>DNBC	ICD-10	Self-reported FA or multivitamin<br>4 weeks prior LMP and 8 weeks after LMP, 4-8 weeks	NA	ASD, n=552<br>Autistic, n=198<br>Asperger's syndrome, n=109<br>PDD-NOS, n=245	Child: sex, birth weight, age<br>Maternal: age, smoking, alcohol consumption, mental, pre-pregnancy BMI, parity, mental illness<br>Paternal: age<br>Household: SES	Folate, ref no use<br>ASD: RR 1.06 (0.82-1.36)<br>Autism: RR 1.18 (0.76-1.84)<br>Asperger's syndrome: RR 0.85 (0.46-1.53)<br>PDD-NOS: RR 1.07 (0.75-1.54)<br>Multivitamin users, ref no use<br>ASD: RR 1.00 (0.82-1.22)<br>Autism: RR 1.22 (0.87-1.69)<br>Asperger's syndrome: RR 0.95 (0.62-1.46)<br>PDD-NOS: RR 0.87 (0.65-1.17)
Schmidt et al. 2012<br>USA	ASD	Case control<br>CHARGE	MSEL<br>ADI-R<br>ADOS	Self-reported FA and multivitamin intake 3 months before and during pregnancy	FA intake calculated based on brand, dose, frequency of consumption product	ASD, n=429<br>DD, n=130<br>TD, n=278	Child: birth year, sex, race<br>Maternal: race, age, educational, pre-pregnancy BMI, birthplace, residing with a smoker, smoking status, alcohol consumption, other nutrient intakes (vitamin A, B_6, C, D, E)	FA, ≥600 µg during first month of pregnancy, OR 0.62 (0.42-0.92) ref <600 µg)<br><br>Among mother and children with MTHFR 677 C>T variant genotypes, OR 0.3 (0.10-0.90)

ABC, Autism Behavior Checklist; ADI-R, Autism Diagnostic Interview-Revised; ADOS, Autism Diagnostic Observation Schedule-Generic; ASD, Autism Spectrum Disorder; BMI; Body Mass Index; CARS, Childhood Autism Rating Scale; CBCL, Child Behavior Checklist; DD, Developmental delay; CHARGE, Childhood Autism Risks from Genetics and Environment; DNBC, Danish National Birth Cohort; DSM, Diagnostic and Statistical Manual of Mental Disorders; EARLI, Early Autism Risk Longitudinal Investigation; FA, Folic Acid; HR, Hazard ratio; HOME, Health Outcomes and Measures of the Environment; ICD, International Classification of Diseases; LMP, Last menstrual period; MARBLES, Markers of Autism Risk in Babies: Learning Early Signs; MoBa, Norwegian Mother and Child Cohort Study; MSEL, Mullen Scales of Early Learning; NA, not available; OR Odds ratio; SES, socioeconomic status; SRS, Social Responsiveness Scale; TD, typical development.

[a]Un-supplemented or very low (median daily dispensed dose<0.2mg, typical of dose levels in women not receiving FA supplementation); low-supplemented (0.2 to <0.4 mg/day); typically-supplemented (0.4 to <1 mg/day, reflecting typical dose levels of women supplementing with commonly available prenatal vitamins); high-supplemented (1 to <3 mg/day) and very high supplemented (>3 mg/day, reflecting clinical recommendations for FA supplementation for some high-risk pregnancies).

[b]Antifolate medication (proguanil, methotrexate, sulfasalazine, sulphamethoxazole and trimethoprim, pyrimethamine, valproate, carbamazepine, phenytoin and phenobarbital)

[c]Anxiety disorder, autism, bipolar disorder, depression, epilepsy, intellectual disability, non-affective psychotic disorders, and stress disorders)

[1]ncludes prenatal, multivitamin, Vit -A, B6, B12, C, D, E, folic acid, iron, calcium, zinc, cereals other supplements: slim fast, instant breakfast, protein powder, brewer’s yeast, st.john’s worts, ginseng, omega-3 fatty acids, fish oil.

Table S2. Odds ratios and 95% CIs for the association between log-transformed maternal serum Vitamin B12 (continuous) and offspring ASD and subtypes adjusting for additional covariates

	**Model I**		**Model II**	
**Log-transformed maternal vitamin B12 levels (pmol/L)**	**Adjusted[a] Odds Ratio (95% CI)**	**P- value**	**Adjusted[b] Odds Ratio (95% CI)**	**P- value**
**ASD**	0.93 (0.79-1.09)	0.406	0.93 (0.79-1.11)	0.423
**Childhood autism**	0.96 (0.72-1.28)	0.764	1.00 (0.75-1.35)	0.989
**Asperger's**	1.04 (0.77-1.40)	0.794	1.00 (0.74-1.36)	0.979
**PDD/PDD-NOS**	0.87 (0.66-1.15)	0.339	0.86 (0.64-1.15)	0.300

ASD, autism spectrum disorder; CI, confidence interval; PDD, Pervasive Developmental Disorder; PDD-NOS, PDD-not otherwise specified.

[a]Adjusted for maternal socioeconomic status, gestational week of blood draw and offspring gestational age.

[b]Adjusted for maternal: history of psychopathology, substance abuse, socioeconomic status, gestational week of blood draw and offspring gestational age.

Table S3: Odds ratios and 95% CIs for the association between maternal serum vitamin B12 levels (in quintiles) and offspring ASD and subtypes adjusting for additional covariates

**Maternal serum vitamin B12 in quintiles (pmol/L)**	**Model I**		**Model II**	
**1. ASD**	**Adjusted[a] Odds Ratio (95% CI)**	**P- value**	**Adjusted[b] Odds Ratio (95% CI)**	**P- value**
0-20 (0-80.8)	1.06 (0.84-1.33)	0.631	1.05 (0.83-1.33)	0.661
21-40 (80.9-105.4)	1.08 (0.86-1.36)	0.492	1.11 (0.88-1.39)	0.388
41-60 (105.5-128.9)	Reference		Reference	
61-80 (128.8-164.9)	1.02 (0.81-1.29)	0.839	1.00 (0.79-1.27)	0.981
≥81 (≥165)	1.01 (0.80-1.27)	0.943	1.03 (0.81-1.30)	0.812
**2. Childhood autism**				
0-20 (0-80.8)	1.49 (0.98-2.27)	0.061	1.53 (0.99-2.35)	0.054
21-40 (80.9-105.4)	1.29 (0.88-1.89)	0.197	1.35 (0.91-1.99)	0.141
41-60 (105.5-128.9)	Reference		Reference	
61-80 (128.8-164.9)	0.96 (0.64-1.45)	0.857	1.00 (0.66-1.52)	0.996
≥81 (≥165)	1.59 (1.06-2.42)	0.027	1.75 (1.14-2.67)	0.010
**3. Asperger's**				
0-20 (0-80.8)	0.89 (0.61-1.29)	0.538	0.87 (0.59-1.27)	0.468
21-40 (80.9-105.4)	1.03 (0.69-1.53)	0.877	1.02 (0.68-1.53)	0.918
41-60 (105.5-128.9)	Reference		Reference	
61-80 (128.8-164.9)	1.27 (0.84-1.89)	0.255	1.19 (0.79-1.82)	0.400
≥81 (≥165)	0.99 (0.67-1.47)	0.970	0.95 (0.63-1.42)	0.802
**3. PDD/PDD- NOS**				
0-20 (0-80.8)	0.87 (0.56-1.33)	0.511	0.86 (0.55-1.34)	0.507
21-40 (80.9-105.4)	0.93 (0.61-1.40)	0.725	0.96 (0.62-1.48)	0.847
41-60 (105.5-128.9)	Reference		Reference	
61-80 (128.8-164.9)	0.88 (0.58-1.35)	0.562	0.83 (0.54-1.29)	0.414
≥81 (≥165)	0.67 (0.44-1.03)	0.067	0.68 (0.44-1.06)	0.086

ASD, autism spectrum disorder; CI, confidence interval; PDD, Pervasive Developmental Disorder; PDD-NOS, PDD-not otherwise specified.

[a]Adjusted for maternal socioeconomic status, gestational week of blood draw and offspring gestational age.

[b]Adjusted for maternal: history of psychopathology, substance abuse, socioeconomic status, gestational week of blood draw and offspring gestational age.

Table S4. Odds ratios and 95% CI of the association between log-transformed maternal serum Vitamin B12 (continuous, quintiles) and offspring autism with and without Intellectual Disability (ID)

**Maternal serum vitamin B12 (pmol/L)**	**Association with offspring autism with ID**					
**Continuous**	**Cases (N=221) Median**	**Controls (N=221) Median**	**Odds Ratio Unadjusted (95% CI)**	**P- value**	**Odds Ratio Adjusted[a] (95% CI)**	**P- value**
Log-transformed analysis	4.75	4.74	1.05 (0.68-1.61)	0.825	1.06 (0.69-1.64)	0.796
**Quintiles**	**n (%)**	**n (%)**	**Odds Ratio Unadjusted (95% CI)**	**P- value**	**Odds Ratio Adjusted[a] (95% CI)**	**P- value**
0-20 (0-80.8)	51 (23.08)	42 (19.00)	1.55 (0.70-3.41)	0.279	1.74 (0.77-3.93)	0.187
21-40 (80.9-105.4)	36 (16.29)	51 (23.08)	0.67 (0.32-1.39)	0.281	0.71 (0.33-1.52)	0.377
41-60 (105.5-128.9)	46 (20.81)	40 (18.10)	Reference		Reference	
61-80 (128.8-164.9)	40 (18.10)	56 (25.34)	0.66 (0.30-1.42)	0.285	0.66 (0.30-1.46)	0.308
≥81 (≥165)	48 (21.72)	32 (14.48)	1.63 (0.73-3.64)	0.232	1.87 (0.80-4.38)	0.148
**Maternal serum vitamin B12 (pmol/L)**	**Association with offspring autism without ID**					
**Continuous**	**Cases (N=1337) Median**	**Controls (N=1337) Median**	**Odds Ratio Unadjusted (95% CI)**	**P- value**	**Odds Ratio Adjusted[a] (95% CI)**	**P- value**
Log-transformed analysis	4.73	4.76	0.88 (0.74-1.05)	0.149	0.92 (0.77-1.10)	0.369
**Quintiles**	**n (%)**	**n (%)**	**Odds Ratio Unadjusted (95% CI)**	**P- value**	**Odds Ratio Adjusted[a] (95% CI)**	**P- value**
0-20 (0-80.8)	280 (20.94)	270 (20.19)	1.54 (0.94-2.52)	0.086	1.42 (0.86-2.34)	0.172
21-40 (80.9-105.4)	292 (21.84)	262 (19.60)	1.64 (1.04-2.57)	0.033	1.58 (0.99-2.49)	0.052
41-60 (105.5-128.9)	244 (18.25)	270 (20.19)	Reference		Reference	
61-80 (128.8-164.9)	270 (20.19)	256 (19.15)	1.23 (0.76-1.99)	0.391	1.09 (0.67-1.78)	0.729
≥81 (≥165)	251 (18.77)	279 (20.87)	1.47 (0.91-2.36)	0.112	1.49 (0.92-2.43)	0.103

CI, confidence interval; Intellectual Disability, ID.
[a]Adjusted for gestational week of blood draw and maternal socioeconomic status.
